# Spatiotemporal Patterns of Loco-Regional Recurrence After Breast-Conserving Surgery

**DOI:** 10.3389/fonc.2021.690658

**Published:** 2021-08-30

**Authors:** Fei-Lin Qu, Rui Mao, Zhe-Bin Liu, Cai-Jin Lin, A-Yong Cao, Jiong Wu, Guang-Yu Liu, Ke-Da Yu, Gen-Hong Di, Jun-Jie Li, Zhi-Ming Shao

**Affiliations:** ^1^Key Laboratory of Breast Cancer in Shanghai, Department of Breast Surgery, Fudan University Shanghai Cancer Center, Shanghai, China; ^2^Department of Oncology, Shanghai Medical College, Fudan University, Shanghai, China

**Keywords:** loco-regional recurrence, breast-conserving surgery, spatiotemporal recurrence pattern, molecular subtype, annual recurrence rate

## Abstract

**Background:**

Loco-regional recurrences (LRR) following breast-conserving surgery (BCS) remain a heterogeneous class of disease that has significant variation in its biological behavior and prognosis.

**Methods:**

To delineate the spatiotemporal patterns of LRR after BCS, we analyzed the data of 4325 patients treated with BCS from 2006 to 2016. Clinico-pathological and treatment specific factors were analyzed using the Cox proportional hazards model to identify factors predictive for LRR events. Recurrence patterns were scrutinized based on recurrence type and recurrence-free interval (RFI). Annual recurrence rates (ARR) were compared according to recurrence type and molecular subtype.

**Results:**

With a median follow-up of 66 months, 120 (2.8%) LRRs were recorded as the first site of failure. Age, pathologic stage, and molecular subtype were identified as predictors of LRR. The major recurrence type was ipsilateral breast tumor recurrence, which mainly (83.6%) occurred ≤5y post surgery. In the overall population, ARR curves showed that relapse peaked in the first 2.5 years. Patients with regional nodal recurrence, shorter RFI, and synchronous distant metastasis were associated with a poorer prognosis. HER2-positive disease had a higher rate of LRR events, more likely to have in-breast recurrence, and had an earlier relapse peak in the first 2 years after surgery.

**Conclusions:**

LRR risk following BCS is generally low in Chinese ethnicity. Different recurrence patterns after BCS were related to distinct clinical outcomes. Management of LRR should be largely individualized and tailored to the extent of disease, the molecular profile of the recurrence, and to baseline clinical variables.

## Introduction

Breast-conserving surgery (BCS) has been established as the standard of care for early-stage breast cancer (EBC). Several large population-based studies have shown that BCS plus radiotherapy is at least equivalent to mastectomy in terms of overall survival (OS) (1–3). However, loco-regional recurrence (LRR) following conservation treatment remains a concern in routine practice, which heralds a poor prognosis and accompanies or precedes distant metastasis in a defined proportion of patients.

The risk of LRR in patients with EBC is primarily assessed by baseline factors. Several clinicopathologic risk factors, including young age at onset, lobular histology, high grade, advanced stage at presentation, and specific molecular subtype, have been shown to be predictive for increased rates of local relapse after BCS and whole-breast radiotherapy (4, 5).

There is a clear need to better identify patients who are at increased risk for LRR despite conventional treatment, and for whom a more tailored locoregional approach could improve outcomes. More difficult questions can arise when scrutinizing the recurrence patterns of LRR with respect to routine clinicopathologic factors. For example, different molecular subtypes are associated with different prognoses, so treatment regimens are typically personalized to the needs of individual patients (6). However, thus far there is minimal data that verifies the association between molecular subtypes and LRR.

Additionally, the evolution over time of the prognosis after LRR has not been well described at the population level. Only two prospective trials have investigated this issue, in which prognosis for patients with LRR is not universally poor, and some subgroups may benefit from adjuvant systemic therapies beyond surgical removal of the LRR. The SAKK 23/82 trial confirmed the beneficial role of tamoxifen in post-recurrence disease-free survival, and the CALOR trial supported the efficacy of chemotherapy for estrogen-receptor negative patients (7, 8). To better inform clinical decision-making, both disease extent and tumor characteristics should be taken into consideration for predicting post-LRR prognosis. The National Comprehensive Cancer Network (NCCN) classifies isolated LRRs into three groups based solely on prior local therapy (9). Given current clinical practices regarding the extent of nodal surgery and regional nodal irradiation, it is perhaps more practical to discuss the management of the breast separate from the nodes.

Therefore, this study aimed to describe different patterns for LRRs manifesting as first-failure events following breast-conserving therapy, using a large institutional population-based registry. First, we present the potential prognostic value of LRR using survival regression model; second, our main objective, the spatiotemporal characteristics of LRR after BCS and finally, the hazard patterns over time according to molecular subtypes.

## Materials and Methods

### Study Population and Ethical Statement

An Institutional Review Board–approved institutional database was used as a source for this analysis. All consecutive patients with invasive breast cancer treated with BCS in 2006 through 2016 were identified.

We selected the following clinicopathological parameters for analysis: age at diagnosis, menopause status, pathologic tumor stage, pathologic node stage, pathology stage, histology, nuclear grade, hormone receptor (HR) status, HER2 status, molecular subtype, systemic treatment information in form of chemotherapy and endocrine therapy. Immunohistochemical (IHC) staining of HR and HER2 status was carried out in the Department of Pathology at our hospital. Lacking data on Ki67 information, the IHC surrogates for 4 mimic subtypes were defined as HR+HER2-, HR+HER2+, HR-HER2+, and HR-HER2- (triple negative). This study was approved by the Ethical Committee of the Shanghai Cancer Center of Fudan University.

### Treatments

The procedure of the BCS technique in our center was described previously (10, 11). Before 2010, the frozen section analysis (FSA) was utilized intraoperatively to evaluate breast margin in our center. Positive surgical margin was defined as tumor (invasive or DCIS) seen immediately at the edge of the resection (12). Since 2010, the inked method has been adopted to alternate the FSA in breast margin management. According to the widely acceptable definition, the use of no ink on tumor is regarded as the standard for an adequate margin in invasive cancer (13). All patients in this database were recommended with radiation therapy (whole-breast radiotherapy with or without regional nodal irradiation). Systemic treatments were administered according to the St. Gallen consensus and NCCN guidelines. In this study, not all patients with HER2-positive disease received 1-year adjuvant trastuzumab treatment because the anti-HER2 targeted drug (Herceptin) was not included in the Catalogue of Drugs for Basic National Medical Insurance until 2017 in China (14).

### Recurrence Definition and Follow-Up

Recurrence events were defined as follows: ipsilateral breast tumor recurrence (IBTR, recurrent tumor occurring after lumpectomy plus radiotherapy in either the breast parenchyma or skin of the ipsilateral breast), regional nodal recurrence (RNR, metastatic disease in the ipsilateral supra/infraclavicular, internal mammary or axillary lymph nodes), and distant recurrence (all other sites of tumor relapse) (15). In the present study, the type of recurrence after lumpectomy included both IBTR and RNR.

IBTR was subdivided into either true local recurrences (TR) or new primary tumors (NP). Patients were considered as NP if the recurrence was distinctly different from the primary tumor with respect to the immunohistochemical-based subtype or the recurrence location was in a different quadrant. TR was defined as the relapses within the same location and similar immunohistochemical-based subtype (16). Synchronous distant metastasis (SDM) was defined as diagnosis within 30 days of an LRR. Metachronous distant metastasis (MDM) was defined as diagnosis beyond the 30-day window (17). In this paper, recurrence-free interval (RFI) signifies time from primary surgical procedure until recurrence in the ipsilateral breast or locoregionally. Other end points, such as loco-regional recurrence-free survival (LRRFS) and OS, were normally defined according to the STEEP System (18). Data was censored as of 30 June 2020, with a median follow-up time of 66 months (range: 6-199 months).

### Statistical Analysis

The Pearson’s χ^2^ test or Fisher’s exact test was used to test associations between categorical variables, and the Wilcox rank-sum test or Kruskal–Wallis test was used to test differences for continuous variables between groups (19). The distribution of OS was estimated using the Kaplan-Meier method. The log-rank test was performed to test the difference in survival between specified subgroups (20). Multivariate analysis was performed by the Cox risk proportion model. The corresponding hazard ratio (HR) was calculated with Fine and Gray’s competing risk regression model (21). The level of significance was set 5% and all *P* values were two-tailed. Annual recurrence rates (ARR) were estimated with a Kernel method of smoothing (22). An R package called bshazard was used to compute the pointwise estimates of the HRs of continuous predictors introduced nonlinearly. Statistical analysis was performed using R version 3.4.1 (http://www.R-project.org) and its appropriate packages and Statistical Package for Social Sciences (version 20.0) software (SPSS Inc., Chicago, IL, USA).

## Results

### Patient and Tumor Characteristics

Overall, 4325 patients were included in this study. The median age at diagnosis was 49 years (range, 17-99 years). Patient demographics, tumor characteristics, and treatment information are noted in **Table 1**. Among the entire population, 62.6% patients had T1 tumors, while 73% were node negative. Of note, 33.1% (229/692) of patients with HER2-positive disease did not receive trastuzumab, while radiotherapy was not administered in 576 (13.3%) patients. A higher overall compliance rate was observed in endocrine therapy compared with that in Herceptin arm (93.0% vs 66.9%, **Supplementary Table 1**).

**Table 1:** Baseline patient and treatment characteristics.

Characteristic	Overall Cohort, No. (%)	LRR cohort, No. (%)
**No. of patients**	4325	120
**Age**		
Mean	49	46.7
Median	49 (17-99)	47 (23-77)
**Age group, years**		
≤50	2599 (60.1)	81 (67.5)
>50-70	1464 (33.8)	30 (25.0)
>70	262 (6.1)	9 (7.5)
**Menopausal status**		
Premenopausal	2459 (56.9)	70 (58.3)
Postmenopausal	1866 (43.1)	50 (41.7)
**Histology**		
Ductal	3870 (89.5)	114 (95.0)
Lobular	71 (1.6)	3 (2.5)
Others	384 (8.9)	3 (2.5)
**Nuclear grade**		
I	134 (3.1)	1 (0.8)
II	2033 (47.0)	42 (35.0)
III	1358 (31.4)	59 (49.2)
Unknown	800 (18.5)	18 (15.0)
**Pathologic T stage**		
T1	2706 (62.6)	70 (58.3)
T2+T3	919 (21.2)	41 (34.2)
Unknown	700 (16.2)	9 (7.5)
**Pathologic N stage**		
N0	3160 (73.0)	73 (60.8)
N1	820 (19.0)	28 (23.3)
N2+N3	204 (4.7)	17 (14.2)
Unknown	141 (3.3)	2 (1.7)
**Pathologic stage**		
I	2102 (48.6)	42 (35.0)
II	1205 (27.9)	51 (42.5)
III	188 (4.3)	15 (12.5)
Unknown	830 (19.2)	12 (10.0)
**ER/PR status**		
ER or PR positive	3251 (75.2)	65 (54.2)
ER and PR negative	992 (22.9)	55 (45.8)
Unknown	82 (1.9)	0 (0)
**HER2 status**		
Amplified	692 (16.0)	46 (38.3)
No amplified	3381 (78.2)	74 (61.7)
Unknown	252 (5.8)	0 (0)
**Molecular subtype**		
HR+HER2-	2670 (61.7)	39 (32.5)
HR+HER2+	447 (10.3)	25 (20.8)
HR-HER2+	245 (5.7)	21 (17.5)
HR-HER2-	707 (16.4)	35 (29.2)
Unknown	256 (5.9)	0 (0)
**Receipt of chemotherapy**		
Yes	1253 (29.0)	95 (79.2)
No	2783 (64.3)	25 (20.8)
Unknown	289 (6.7)	0 (0)
**Receipt of radiotherapy**		
Yes	3311 (76.6)	84 (70.0)
No	576 (13.3)	36 (30.0)
Unknown	438 (10.1)	0 (0)
**Receipt of anti-HER2 therapy**		
Yes	463 (10.7)	12 (10.0)
No	3754 (86.8)	108 (90.0)
Unknown	108 (2.5)	0 (0)
**Receipt of endocrine therapy**		
Yes	2898 (67.0)	51 (42.5)
No	1067 (24.7)	69 (57.5)
Unknown	360 (8.3)	0 (0)

LRR, loco-regional recurrence; ER, estrogen receptor; PR, progesterone receptor; HR, hormone receptor.

**Figure 1:**
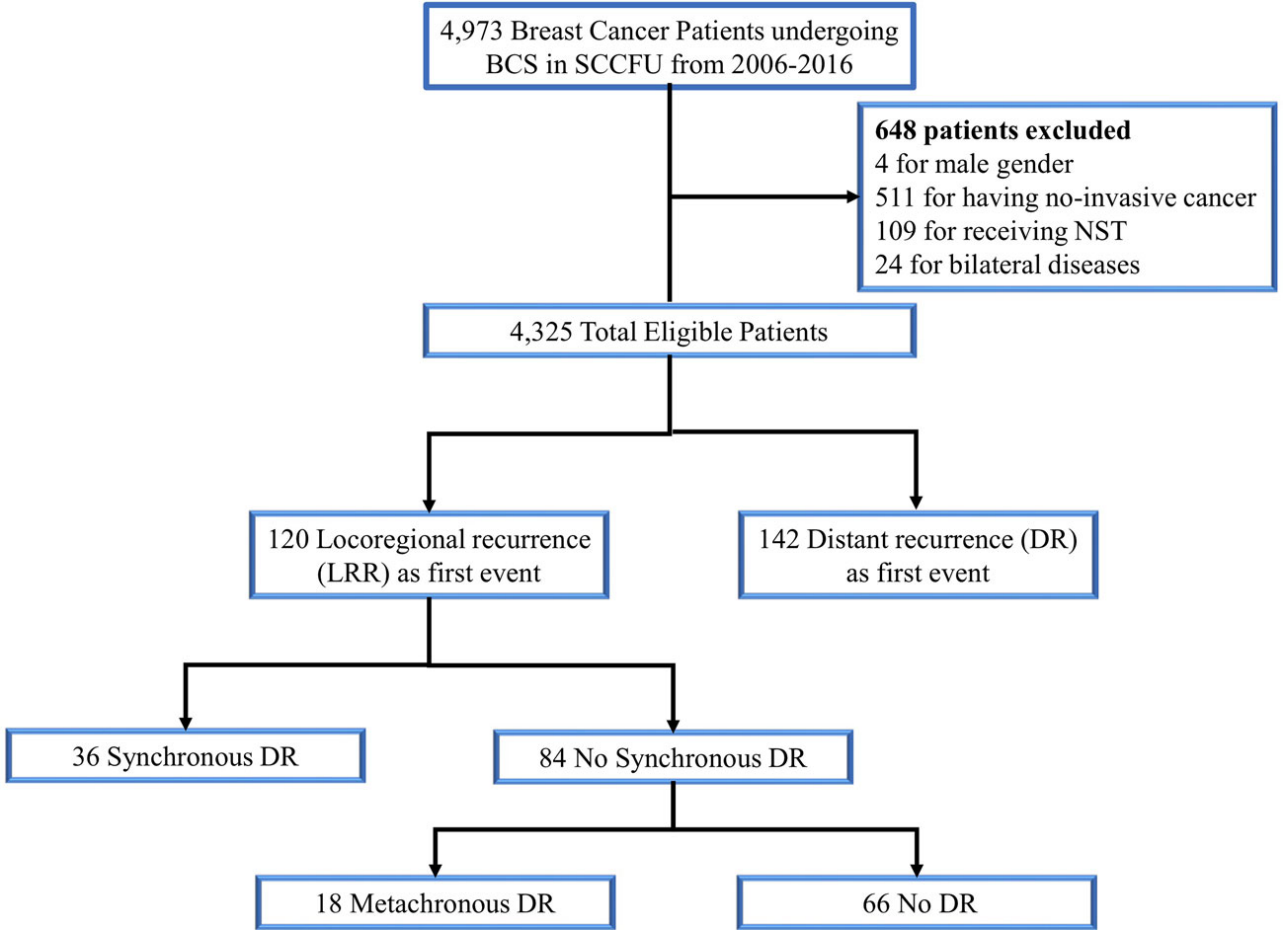
Consort diagram for the study cohort. SCCFU, Shanghai cancer center of Fudan university; BCS, breast-conserving surgery; NST, neoadjuvant systemic therapy; DR, distant recurrence.

With a median follow-up of 66 months (range: 6-199 months), 120 (2.8%) cases of LRR and 142 (3.3%) cases of distant relapse were recorded as a first failure (**Figure 1**). In the overall population, the loco-regional failure rate was low, accounting for a 5-year LRRFS of 97.0% (95%CI= 96.5-97.6). Additionally, among the patients that experienced LRR, 30% (36/120) were diagnosed with SDM, while 18 (15%) patients developed MDM during the study period.

### Factors Associated With LRR and Overall Prognostic Significance

The uni- and multivariate associations between each clinicopathological variable and LRR are presented in **Table 2**. In detail, the factors associated with LRR in the final multivariable model were age ≤45 years (*P* = 0.038; HR [95% CI]= 1.49 [1.02–2.17]), stage II (*P <*0.001; HR [95% CI]= 2.03 [1.34-3.09]) or stage III disease (*P <*0.001; HR [95% CI]= 3.91 [2.12–7.24]), HR+HER2+ (*P <*0.001; HR [95% CI]= 4.18 [2.32-7.52]), HR-HER2+ (*P* =0.014; HR [95% CI]= 3.19 [1.26-8.06]) or HR-HER2- (*P <*0.001; HR [95% CI]= 5.21 [4.54-9.68]) subtypes, no receipt of radiation therapy (*P* =0.015; HR [95% CI]= 2.93 [1.90-4.54]), no administration of anti-HER2 therapy (*P* =0.015; HR [95% CI]= 2.25 [1.17-4.33]) and no use of endocrine treatment (*P* =0.003; HR [95% CI]= 2.84 [1.43-5.64]).

**Table 2:** Factors predictive of loco-regional recurrence in patients treated with breast-conserving surgery.

		Univariable analysis			Multivariable analysis			
		HR (95% CI)		*P* Value	HR (95% CI)	*P^a^* Value		
**Age, years**								
≤45		1.41 (0.98-2.00)		.062	1.49 (1.02-2.17)	.038*		
>45		Ref			Ref			
**Menopausal status**								
Premenopausal		Ref			Ref			
Postmenopausal		0.91 (0.51-1.61)		.735	0.71 (0.39-1.27)	.249		
**Histology**								
IDC		Ref			Ref			
ILC		1.58 (0.50-4.99)		.432	1.22 (0.34-4.37)	.756		
Others		0.24 (0.08-0.74)		.014	0.20 (0.06-0.72)	.013		
**Grade**								
G1/G2					Ref			
G3		2.30 (1.55-3.39)		.001	1.21 (0.78-1.89)	.390		
Unknown		1.14 (0.67-1.93)		.627	1.55 (0.82-2.93)	.174		
**Pathological stage**								
Stage I		Ref			Ref			
Stage II		2.13 (1.42-3.20)		.001	2.03 (1.34-3.09)	.001*		
Stage III		4.15 (2.30-7.46)		.001	3.91 (2.12-7.24)	.001*		
Unknown		0.82 (0.45-1.50)		.516	0.81 (0.44-1.49)	.478		
**Molecular subtype**								
HR+/HER2-		Ref			Ref			
HR+/HER2+		3.85 (2.36-6.28)		.001	4.18 (2.32-7.52)	.001*		
HR-/HER2+		5.59 (3.25-9.62)		.001	3.19 (1.26-8.06)	.014*		
HR-/HER2-		3.42 (2.19-5.33)		.001	5.21 (4.54-9.68)	.001*		
Unknown		0 (0-Inf)		.993	0 (0-Inf)	.993		
**Chemotherapy**								
Yes		Ref			Ref			
No		0.48 (0.29-0.77)		.003	0.94 (0.54-1.64)	.827		
Unknown		0.37 (0.12-1.18)		.093	0.38 (0.10-1.50)	.168		
**Radiotherapy**								
Yes		Ref			Ref			
No		2.88 (1.95-4.26)		.001	2.93 (1.90-4.54)	.015*		
Unknown		0.94 (0.45-1.95)		.870	1.28 (0.51-3.24)	.601		
**Anti-HER2 therapy**							
Yes		Ref			Ref			
No		0.61 (0.36-1.02)		.067	2.25 (1.17-4.33)	.015*		
Unknown		0.33 (0.08-1.42)		.136	0.61 (0.12-3.08)	.546		
**Endocrine therapy**								
Yes		Ref			Ref			
No		3.62 (2.51-5.20)		.001	2.84 (1.43-5.64)	.003*		
Unknown		1.17 (0.50-2.72)		.715	1.70 (0.62-4.65)	.301		

IDC, invasive ductal carcinoma; ILC, invasive lobular carcinoma; HR, hormone receptor.

a Significance of hazard ratio (HR) was calculated with Fine and Gray’s competing risk regression model. The bold values and “*” symbol mean the difference is statistically significant.

**Figure 2:**
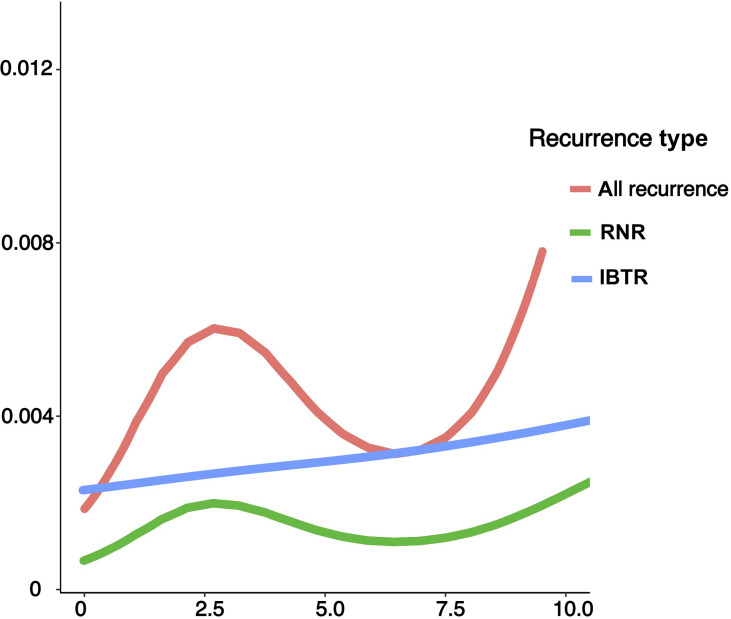
Annual recurrence rates of loco-regional recurrence after lumpectomy in the entire population and categories of different types of recurrence. IBTR, ipsilateral breast tumor recurrence; RNR, regional nodal recurrence.

A graphical representation of ARR curves were shown in **Figure 2**. In the overall population, the curve followed a unimodal distribution, with a clear peak occurring near the 2~3-year intervals and a constantly growing trend thereafter. Specified by type of recurrence, the HR curve in RNR exhibited one peak near 2-3 years, corresponding to that observed in the entire population. In contrast, IBTR did not demonstrate an obvious recurrence surge, but rather presented a continually increasing risk.

### Spatiotemporal Loco-Regional Recurrence Patterns Post-Lumpectomy

**Figure 3:**
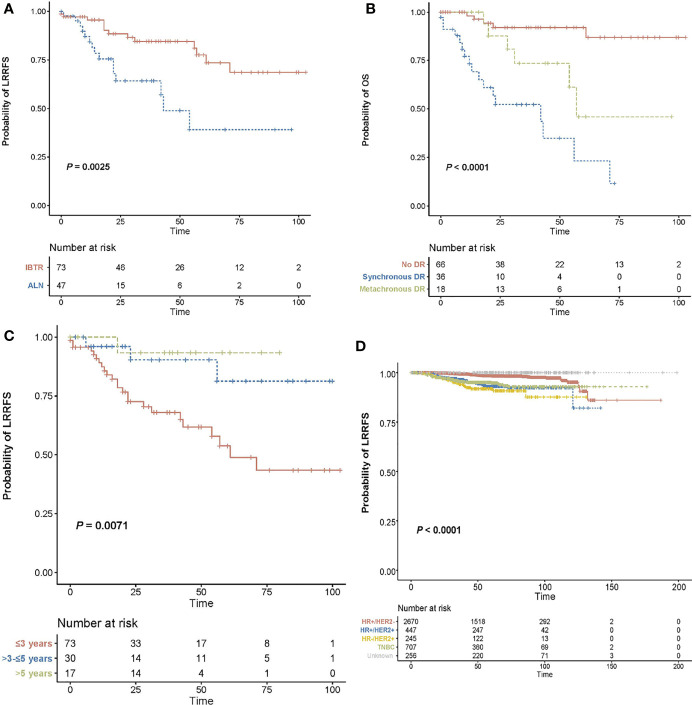
Overall survival curves after lumpectomy. **(A)** Kaplan-Meier curves for overall survival in patients with (either as synchronous or subsequent failures) and without distant recurrence in the setting of loco-regional recurrence. **(B)** Kaplan-Meier curves for overall survival based on recurrence type. **(C)** Kaplan-Meier curves for overall survival according to recurrence-free interval. **(D)** Kaplan-Meier curves for loco-regional recurrence-free survival specified by molecular subtype. ]OS, overall survival; LRRFS, loco-regional recurrence-free survival; DR, distant recurrence; IBTR, ipsilateral breast tumor recurrence; RNR, regional nodal recurrence.

**Figure 3** provides an overview of OS curves after lumpectomy. OS was worse for patients with RNR than for those with in-breast recurrence (5-year OS: 39.1% vs 77.6%; *P* = 0.0025; **Figure 3A**). Notably, worse OS was observed for patients with distant metastases, either SDM or MDM, compared with those who did not experience a distant recurrence (3-year OS: 52.3% vs 73.4% vs 91.9%; *P* = 0.001; **Figure 3B**). Moreover, a significant difference in OS was observed between early and late recurrence. **Figure 3C** confirmed that patients with early recurrences (3 years plus 3-5 years post surgery), had a significant lower rate of OS than patients without relapse until 5 years after primary surgery (overall log-rank *P* = 0.025).

Spatiotemporal characteristics of LRR were documented in **Table 3** specified in recurrence type and time-to-recurrence. In terms of the spatial location of 120 LRR cases, 61.8% (73/120) patients experienced recurrence within the ipsilateral breast, and 47 cases (39.2%) presented with regional lymph node involvement. Using the classification scheme outlined above, 37 cases of in-breast recurrence were classified as TR, 22 were classified as NP and 14 were unable to be classified. NP patients had a longer interval to breast relapse than TR patients (62.6 months vs. 39.4 months, *P*<0.001, **Supplementary Table 2**). Analysis of time distribution of LRR after lumpectomy revealed that 60.8% (73/120) of recurrence events were detected within 3 years post surgery, 25% (30/120) were reported within 3-5 years, and 14.2% (17/120) were reported after 5 years. Considering the recurrence type and recurrence time together, there was no significant difference in distribution of time frame between IBTR and RNR (*P* = 0.670; **Table 3**). Furthermore, SDM occurred in 6.8% of patients initially detected with local disease versus 59.6% who suffered regional disease. Patients with in-breast recurrence were more probable to remain without distant metastases, while those with regional relapse tended to have SDM (*P <*0.001, respectively).

**Table 3:** Spatiotemporal recurrence patterns across different categories.

N=120	LRR location site		Total	*P V*alue
	IBTR	Regional recurrence		
**RFI**				0.670
** ≤3 yrs**	43	30	73	
** 3-5 yrs**	18	12	30	
** >5 yrs**	12	5	17	
**Type of first DM**				.001*
** No DM**	52	14	66	.001*
** SDM**	5	28	33	.001*
** MDM**	16	5	21	.112

LRR, loco-regional recurrence; IBTR, ipsilateral breast tumor recurrence; RFI, recurrence-free interval; DM, distant metastasis; SDM, synchronous distant metastasis; MDM, metachronous distant metastasis.

*P **<**.0167 was set as level of significance by Partitions of Chi-Square method. The bold values mean the difference is statistically significant.

**Figure 4:**
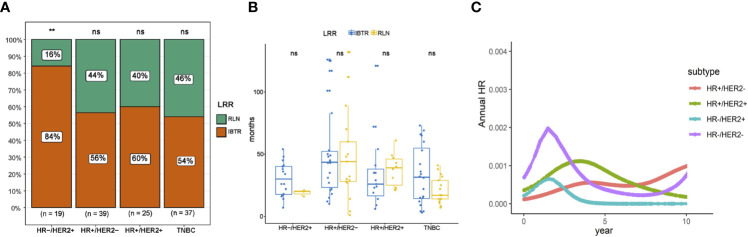
Patterns of recurrence based on molecular subtype. **(A)** Distribution of recurrence type on the basis of molecular subtype. **(B)** Comparison of recurrence-free interval between different recurrence types on the basis of molecular subtype. **(C)** Annual recurrence rates of loco-regional recurrence specified by molecular subtype. IBTR, ipsilateral breast tumor recurrence; RLN, regional lymph node; NS, no significance. The “**” symbol means the difference is statistically significant. The expanded form of "ns" means the difference is not statistically significant.

Given the impact of tumor biology on the pattern of recurrence, we performed an exploratory analysis according to molecular subtype classification. LRRs occurred most frequently in case of HR-HER2+ disease (7.5%), compared to 6.9% in HR+HER2+, 4.7% in triple negative, and 1.2% in HR+HER2-disease, respectively (**Figure 3D**; adjustment by treatment period of Herceptin in **Supplementary Figure 1**). In the context of LRR, HR-HER2+ disease was more likely to have in-breast recurrence than regional recurrence, compared with the three other subtypes. However, no difference in RFI was observed between recurrence types based on molecular subtypes (**Figures 4A, B**). Additionally, we explored the time-varying pattern of LRR according to specific molecular subtypes. For HR-negative (HR-) diseases, a clear sharp surge was remarkably noted at the timepoint of 2 years after initial tumor removal, in which the triple negative group exhibited a higher ARR. Thereafter both HR-HER2+ and triple negative groups rapidly decline to a lower hazard. When it comes to HR-positive (HR+) diseases, the hazard rate manifested a less prominent and more stable pattern with a delayed recurrence peak at nearly 4 years after surgery, in which HR+HER2+ disease showed a higher hazard of recurrence. Notably, a late peak effect is distinctly observed in HR+HER2- subtype, which exhibited a persistent and increasing risk of relapse, leaving a wide plateau in the middle region spanning from about 4 to 7 years and reaching its peak at approximately 9-10 years (**Figure 4C**).

## Discussion

This study is, to the best of our knowledge, the first retrospective study to delineate spatiotemporal patterns of LRR in a Chinese population. Distinct patterns of LRR resulted in different clinical outcomes.

We verified that IBTR and RNR are distinct entities of LRR with different survival prognoses. Variation according to recurrence type is consistent with clinical acumen because RNR can be perceived as a prelude to more-aggressive disease, due to the high risk of concomitant distant relapses. In contrast, in-breast recurrence refers to a limited event with a fairly favorable prognosis. Therefore, guidelines recommend treating patients with in-breast recurrences with standard salvage mastectomy, while in the case of nodal recurrence, a multimodal strategy should be adopted that includes systemic treatment by means of chemotherapy and/or endocrine therapy, in addition to vigorous locoregional treatment (23, 24).

We additionally found that 55% (66/120) of patients with LRR manifested as local relapse alone and demonstrated a satisfactory OS, suggesting that, in a certain proportion of patients, LRR represents a relatively local process, warranting the preferential use of salvage surgical procedures. However, 45% (54/120) of patients with LRR were identified with distant recurrence events synchronously or thereafter. Moreover, patients with these locoregional events experienced poorer OS, particularly for those with SDM. Thus, intensified systemic treatment strategies should be adopted in locoregional events that are at increased risk of developing distant metastases. These results are consistent with those from previous studies and demonstrate that LRR has significant variation in its prognosis and preferred treatments (25). For patients with initial LRR, the detection of concomitant distant disease determines whether the intention of treatment is curative or palliative. Therefore, full preoperative restaging is imperative for optimal treatment planning (26).

There is an unmet need of surrogates for distinguishing “late recurrences” from “early recurrences” in the clinical course of tumor relapse. The recurrence-free interval is a commonly used end point to stratify time frame. In line with results from previous studies (15, 27), our findings emphasized that late-recurring patients (after 5 years) had worse outcomes compared to those with early recurrences (within 3 years). However, one limitation that our study shared with the above-mentioned studies is the arbitrary cut-off threshold of RFI for identifying early and late recurrence. Recurrence 5 years or more after surgery is universally referred to as late recurrence and account for nearly one-half of all recurrence events in HR-positive disease (28). Of note, we observed a significant difference in OS between groups with so-called “early recurrences” (3 years versus 3-5 years post surgery). This result suggests that well-accepted “5 years after surgery” is a time point that does not precisely discriminate subsets with good and poor prognoses. The accurate definition of LRRs as “early” or “late” recurrence is imperative in the setting of recurrence to serves as a benchmark in prognostic evaluations and therapeutic decision aides.

Molecular subtypes in breast cancer have been correlated with differences in LRR and OS. The lowest LRR rates we observed were in HR+HER2- breast cancer, which was consistent with results from previously published studies (29, 30). In addition, worse local control was confirmed in triple negative and HER2-positive diseases compared with the other subtypes, as has also been previously reported (31, 32). In the current study, an exploratory analysis according to molecular subtypes was carried out to investigate the influence of biology subtype on recurrence patterns. HR-HER2+ disease was found to be more likely to have in-breast recurrence than regional nodal recurrence, compared with the other three subtypes. However, no difference in time-to-recurrence was observed between in-breast recurrence and regional nodal recurrence, regardless of molecular subtype.

By using ARR to analyze dynamic pattern in recurrence risk by year, we verified a unimodal time distribution of recurrence risk in women with HR+HER2+, HR-HER2- and triple negative subtypes; however, in patients with HR+HER2- disease, a bi-modal peak pattern of hazard rate was displayed with a first peak at 4 years and a second peak at 9-10 years. Importantly, we showed that the early peak (2 years after surgery) was most pronounced in triple negative subgroup, and is likely to be attributed to the aggressive and metastatic behaviors of this subtype. The late recurrences in HR+HER2- disease seemed to be approximately ascribed to distant recurrences, which might possibly give further support to a previously unknown dormancy state that, at the primary tumor surgical removal, results in evolving chemo-sensitive metastatic processes, and, moreover, of a later chemo-refractory dormancy state (33). To sum up, the monitoring and follow-up strategy for LRR should be scheduled individually based on different subtypes.

The main strength of this study includes the comprehensive overview of annual incidence rates of LRR and subsequent outcome post-LRR in conservatively treated women diagnosed with an invasive EBC. Furthermore, an active follow-up program was conducted by FUSCC database staff with a completion rate for 99% of all enrolled patients, in which all recurrent cases (LRR and distant metastasis) that occurred within 10 years after hospital discharge were registered, thereby ensuring the reliance on level of accuracy. To the best of our knowledge, this is the largest retrospective analysis of post-conservation recurrence patterns for breast cancer patients in a Chinese population.

We acknowledge that the present study has inherent limitations. Primarily due to the monocentric retrospective nature of this study, the administrated treatments might have been biased by institutional practice procedures, which do not necessarily reflect the actual standards in terms of endocrine therapy and chemotherapy. Secondly, systemic targeted therapy has more importance on the nature of HER2-positive tumors regardless of the provision of prior local treatment. Anti-HER2 drugs of trastuzumab was introduced to Chinese medical insurance until 2017, therefore, not all HER2+ patients received targeted treatment in this cohort. Our results that HER2-positive breast cancers were associated with the lowest LRRFS should be approached cautiously. Furthermore, lack of Ki-67 information impeded us from further classification of luminal A and B subtypes.

In conclusion, LRR risk following BCS is generally low among Chinese patients with EBC. Different recurrence patterns after lumpectomy resulted in distinct prognoses. Management of LRR should be largely individualized and tailored to the extent of the disease, the molecular profile of the recurrence, and baseline clinicopathologic factors, all of which should also inform new guidelines for breast cancer follow-up and surveillance.

## Data Availability Statement

The raw data supporting the conclusions of this article will be made available by the authors, without undue reservation.

## Ethics Statement

The studies involving human participants were reviewed and approved by Ethical Committee of Shanghai Cancer Center of Fudan University. Written informed consent for participation was not required for this study in accordance with the national legislation and the institutional requirements.

## Author Contributions

Conceptualization: F-LQ, RM, Z-BL, J-JL, and Z-MS. Data curation: F-LQ, RM, Z-BL, C-JL, J-JL, and Z-MS. Formal analysis: F-LQ, RM, and C-JL. Project administration, resources, and software: A-YC, JW, G-YL, K-DY, and G-HD. Writing – original drafting: F-LQ, RM, and Z-BL. Writing – review and editing: J-JL and Z-MS. All authors contributed to the article and approved the submitted version.

## Conflict of Interest

The authors declare that the research was conducted in the absence of any commercial or financial relationships that could be construed as a potential conflict of interest.

## Publisher’s Note

All claims expressed in this article are solely those of the authors and do not necessarily represent those of their affiliated organizations, or those of the publisher, the editors and the reviewers. Any product that may be evaluated in this article, or claim that may be made by its manufacturer, is not guaranteed or endorsed by the publisher.

## Supplementary Material

Supplementary Figure 1 Local control after lumpectomy specified by treatment period based on drug availability (Herceptin). **(A)** Kaplan-Meier curves for loco-regional recurrence-free survival specified by molecular subtype in pre-Herceptin treatment era (2006-2014). **(B)** Kaplan-Meier curves for loco-regional recurrence-free survival specified by molecular subtype in post-Herceptin treatment era (2015-2016).
