# Supplementary figures and images for: Spatiotemporal Patterns of Loco-Regional Recurrence After Breast-Conserving Surgery
Source: Front Oncol. 2021 Aug 30;11:690658. doi: 10.3389/fonc.2021.690658 (PMC8435899; doi:10.3389/fonc.2021.690658)

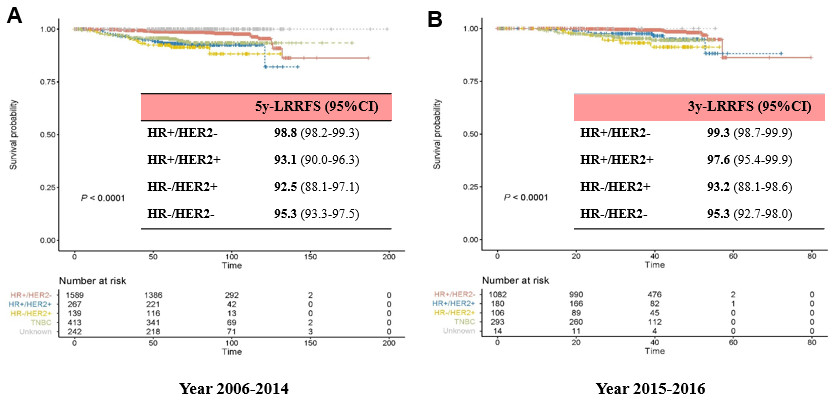

Supplement: Supplementary Figure 1 — Local control after lumpectomy specified by treatment period based on drug availability (Herceptin). (A) Kaplan-Meier curves for loco-regional recurrence-free survival specified by molecular subtype in pre-Herceptin treatment era (2006-2014). (B) Kaplan-Meier curves for loco-regional recurrence-free survival specified by molecular subtype in post-Herceptin treatment era (2015-2016). [file Image_1.jpeg]
