# Supplementary material for: Spatiotemporal Patterns of Loco-Regional Recurrence After Breast-Conserving Surgery
Source: Front Oncol. 2021 Aug 30;11:690658. doi: 10.3389/fonc.2021.690658 (PMC8435899; doi:10.3389/fonc.2021.690658)
Supplement: Supplementary file 2 [file Table_1.docx]

**Table S1. Patient compliance with Herceptin and endocrine therapy**

| **IHC-based subtype** | **Herceptin compliance** | | **EnT Compliance** | |
| --- | --- | --- | --- | --- |
|  | ***N*** | ***%*** | ***N*** | ***%*** |
| **HR+HER2- (*N*=2670)** | **/** | **/** | 2498 | 93.6 |
| **HR+HER2+ (*N*=447)** | 285 | 63.8 | 400 | 89.5 |
| **HR-HER2+ (*N*=245)** | 178 | 72.7 | **/** | **/** |

**IHC**, immunohistochemistry; **HR**, hormone receptor; **EnT**, endocrine therapy.

**Table S2. Clinical characteristics of 59 in-breast recurrence by classified categories**

| **Characteristic** | **True recurrence**  **(n=37)** | **New Primary**  **(n=22)** | ***P V*alue** |
| --- | --- | --- | --- |
| **Mean age at diagnosis** | 48.7±1.4 years | 47.9±1.6 years | 0.42 |
| **Mean age at relapse** | 52.2±1.8 years | 56.2±1.7 years | 0.12 |
| **Mean time to relapse** | 39.4 months | 62.6 months | ***.001**** |

**P* ***<*** .05 was set as level of significance.
